# Use of Lot quality assurance sampling surveys to evaluate community health worker performance in rural Zambia: a case of Luangwa district

**DOI:** 10.1186/s12913-017-2229-9

**Published:** 2017-04-17

**Authors:** Moses Mwanza, Japhet Zulu, Stephanie M. Topp, Patrick Musonda, Wilbroad Mutale, Roma Chilengi

**Affiliations:** 1 Centre for Infectious Disease Research in Zambia, Plot No. 5032, Great North Road, P.O. Box 34681 Lusaka, Zambia; 2 University of North Carolina at Chapel Hill, Chapel Hill, NC USA; 3 University of Zambia, School of Medicine, Lusaka, Zambia; 4 University of Alabama at Birmingham, Birmingham, AL USA

**Keywords:** Lot quality assurance sampling, Community Health Worker performance, Quarterly surveys, Rural Zambia

## Abstract

**Background:**

The Better Health Outcomes through Mentoring and Assessment (BHOMA) project is a cluster randomized controlled trial aimed at reducing age-standardized mortality rates in three rural districts through involvement of Community Health Workers (CHWs), Traditional Birth Attendants (TBAs), and Neighborhood Health Committees (NHCs). CHWs conduct quarterly surveys on all households using a questionnaire that captures key health events occurring within their catchment population. In order to validate contact with households, we utilize the Lot Quality Assurance Sampling (LQAS) methodology. In this study, we report experiences of applying the LQAS approach to monitor performance of CHWs in Luangwa District.

**Methods:**

Between April 2011 and December 2013, seven health facilities in Luangwa district were enrolled into the BHOMA project. The health facility catchment areas were divided into 33 geographic zones. Quality assurance was performed each quarter by randomly selecting zones representing about 90% of enrolled catchment areas from which 19 households per zone where also randomly identified. The surveys were conducted by CHW supervisors who had been trained on using the LQAS questionnaire. Information collected included household identity number (ID), whether the CHW visited the household, duration of the most recent visit, and what health information was discussed during the CHW visit. The threshold for success was set at 75% household outreach by CHWs in each zone.

**Results:**

There are 4,616 total households in the 33 zones. This yielded a target of 32,212 household visits by community health workers during the 7 survey rounds. Based on the set cutoff point for passing the surveys (at least 75% households confirmed as visited), only one team of CHWs at Luangwa high school failed to reach the target during round 1 of the surveys; all the teams otherwise registered successful visits in all the surveys.

**Conclusions:**

We have employed the LQAS methodology for assurance that quarterly surveys were successfully done. This methodology proved helpful in identifying poorly performing CHWs and could be useful for evaluating CHW performance in other areas.

**Trial registration:**

Identifier: NCT01942278. Date of Registration: September 2013.

**Electronic supplementary material:**

The online version of this article (doi:10.1186/s12913-017-2229-9) contains supplementary material, which is available to authorized users.

## Background

Although most low income countries including Zambia have adopted Primary Health Care (PHC), access to basic health care still remains a challenge [1, 2]. The WHO defines PHC as essential community-based health care that is universally accessible to individuals, families, groups, communities, and populations, is driven by community participation in identifying health issues and making decisions on appropriate solutions, and is sustained by the community [2, 3]. This approach often involves utilization of Community Health Workers (CHWs) as a community-based resource to address the immediate shortage of professional health workers [3]. This shortage of human resources is worst in rural areas, resulting in greater morbidity and mortality in rural communities [1, 4, 5].

Acknowledging the shortage of formally trained health workers, the Zambian National Health Strategy presently allows for standard training of volunteer CHWs to deliver basic community-based primary health care [5, 6]. Recently, the government embarked on a programme to provide formal training of CHWs for 1 year, after which they are employed by the government as part of the formally recognized health work force and return to serve their respective communities [6]. This approach is not unique to Zambia as many other low-middle income countries are also heavily dependent on CHWs to provide health services, especially in rural areas [6, 7]. Evidence suggests that this cadre of health workers is being used by several nongovernmental organizations (NGOs) and civil societies globally [7–9].

To respond to the human resource challenges in rural Zambia, the Centre for Infectious Disease Research in Zambia (CIDRZ) has been implementing a large health systems strengthening programme called Better Health Outcomes through Mentoring and Assessments (BHOMA), which leverages CHWs to improve service delivery [10]. Through BHOMA, CHWs were trained to work in the Out Patient Department (OPD) to screen patients and perform simple procedures such as checking vital signs and initiating patient record files. Some of the CHWs were trained to follow-up patients in the community for which they were trained on use of mobile phones for real-time electronic data capture and transmittal over the regular Global System for Mobile communications (GSM) network, which has been reported elsewhere [11]. The BHOMA data system tracks patients presenting to the OPD with clinical “danger signs” and for those who do not return on appointed review dates. Reminder text messages were sent to the respective CHW covering the communities where the patient registered as a permanent residence when giving their demographic information during household visits and at the health facility registration department. The CHWs also undertake quarterly cross-sectional household visits collecting key health events within their catchment population. Each health facility has a CHW supervisor to oversee activities and ensure that appropriate roles and tasks are achieved [10, 11]. The use of mobile phones elsewhere by CHWs has proven to improve community case management and collection of complete, timely, and precise health data for future research in rural areas of Africa [11].

To provide quality assurance on CHW performance, the BHOMA project employed the Lot Quality Assurance Sampling (LQAS) methodology [12]. LQAS was originally developed to control the quality of output in industrial production processes and later on used for conducting health surveys [13, 14]. LQAS has emerged as a useful tool in public health to identify low-performing program areas and to monitor developing countries’ health programs at different levels of the health care delivery system [13–15]. In Rwanda, LQAS was successfully used for data quality assessment of the CHW program in the documentation of key demographic and health indicators leading to improved quality of data collection [16].

This paper reports application of the LQAS approach to monitor CHW performance in relation to completing quarterly household surveys and health information sharing with the target communities, in Luangwa district, which is one of rural districts in Zambia.

## Methods

Between April 2011 and December 2013, seven health facilities in Luangwa District were enrolled into the BHOMA project LQAS survey study. Each health facility catchment area was divided into geographical zones, where each zone included up to a maximum of 300 households. Existing and new CHWs from within the communities were recruited through established community participatory methods by engaging traditional leaders and neighborhood health committee members from all villages within the zone to ensure representativeness for all zones. Using the existing structures in each community, both existing and new CHWs were recruited following open advertisement and interviews. Selected CHWs met minimum qualifications of reaching grade 7 and ability to read and write.

A total of 70 candidate CHWs were interviewed from which 33 were recruited according to the number of the district zones for the study. Of these 14 were female (age range 18 to 35) and 19 were male (age range 20 to 48). The median educational level among CHWs was grade 12 (range 7–12); see Table 1.

**Table 1:** CHW survey performance by round, gender and educational level of responsible CHW

Site	Zone	Survey1	Survey2	Survey3	Survey4	Survey5	Survey6	Survey7	Sex	Education. Qualification
Mandombe	1	19	19	19	19	19	19	19	M	9
	2	19	19	19	19	19	19	19	M	12
	3	19	19	19	19	19	19	19	F	12
	4	19	19	19	18	18	19	19	F	12
Luangwa Boma	4	19	18	18	18	18	17		F	12
	2	17		17			17	19	F	12
	5	18	15	17	17	17	18	19	F	12
	6	19	18						M	9
	1		18	15	19	19		19	F	9
	3				19	19	19	19	F	9
Mphuka	3	19	18	17			19	3	F	9
	1	19		19	19	19	19	19	M	12
	5	19	18	17			19	19	M	9
	2	19	19	18	19	19	19	19	M	9
	4	19	19	19	19	19	19	19	M	9
Kasinsa	3	17	15	19	18	18	19	18	M	12
	2	19	18	17	17	17	18	18	M	9
	1	18	17	19			19	19	M	12
	5	14	17	16			15	19	M	12
	4			17	18	18	19	19	F	12
Luangwa High Sch.	4	11	18	17	16	16	19	19	F	9
	2	8	17	17	19	19	18	19	M	12
	3	13	18	19	19	19	19	19	M	12
	1	11	19	18	19	19	19	19	M	12
Sinyawagora	4				16	16	19	18	M	9
	1				17	17	19	19	F	9
	3				17	17	19	19	F	9
	2				17	17	19	19	F	9
Chitope	2						19	19	M	7
	4						19	19	M	12
	5						18		M	12
	6						19	19	M	12
	3							19	F	12
TOTAL HH VISITED PER ROUND		355	358	392	398	398	558	551	3010	
TOTAL EXPECTED HH TO BE VISITED PER ROUND		399	380	418	418	418	570	570	3173	

The CHWs underwent project-specific orientation on a “community care” package. The training package included data entry using mobile phones and collection of demographic data, age bands, tracking and recording of mortalities. They were also taught on how to collect data on HIV, pregnancy, immunizations, and were to conduct various health-related topics. CHWs conducted monthly household surveys with the goal of reaching all households in their respective zones each quarter and thereafter submit electronically the data collected to the data management team based at the BHOMA central office in Lusaka. If they found a patient with a life-threatening condition or a clinical “danger sign”, they were trained to provide start doses of basic drugs from the CHW drug kit such as paracetamol and oral rehydration salts and then refer the patient to the nearest heath centre. Clinical “danger signs” include failure to drink or breastfeed, continuous vomiting, convulsions, lethargy/unconsciousness, chest in-drawing, severe shortness of breath, severe bleeding, and severe palmer pallor. The danger signs were classified according to the Zambia Ministry of Health integrated management of childhood illnesses (IMCI) classification guidelines. Pregnant women who had not started antenatal care were also referred to the maternal child health facility.

As part of quality assurance process, CHW supervisors were trained to coordinate the activities of CHWs and to use the LQAS survey questionnaire (see Additional file 1) to confirm CHW household visitation. Based on the households visited by CHWs in the previous quarter, the data management team at the BHOMA central office in Lusaka generated a random list of sampled households according to LQAS methodology [13].

The BHOMA study was reviewed and approved by both the University of Zambia Biomedical Ethics and the University of North Carolina at Chapel Hill Ethics Committees.

### Data collection

In each of the selected zone, 19 households were sampled by the CHW supervisors using a standardized questionnaire to be completed by either the head of the household or any member above 16 years as listed in Additional file 1. A sample size of 19 provides an acceptable level of error for decision-making by managers; at least 90% of the time, it identifies areas that have reached the set target or below the average coverage of the programme [17]. Since LQAS uses the binomial formula to calculate smaller samples and to come up with a decision criteria for grouping CHWs by their performance using a three triage assessment system; adequate, inadequate and very inadequate [15]. In this study, the same criteria was used to assign the CHW to the triage system and these were; coverage as been adequate if 75% or more of the targeted households were visited, inadequate if the coverage was between 50 and 75% and very inadequate if 50% or less. For instance in a sample of 19 households if only five or fewer have not been visited, then the CHW is said to have provided adequate coverage. If more than five households have not been visited, the CHW performance is considered as inadequate.

The CHW supervisors validated the data by counterchecking the unique household identity number and a summary of the health information discussed. Once the questionnaires were completed, they were submitted to the district study team for further data completeness checks before forwarding the forms to the data management team at the central office in Lusaka. CHW coverage performance was assessed by confirming physical visitation of a CHW to each household listed on the quarterly sample within each zone and delivery of health information on at least one health topic. A threshold was set apriori at 75% as the minimum standard for coverage.

### Data analysis

To use LQAS decision rules, the study applied two rules for analysis;

1. Define the performance standards for the survey coverage under the study using the three-part triage system.
2. Develop a decision rule that states the maximum number of households which have not been visited during the intervention allowed in the LQAS. Any number greater than this threshold results in a CHW performance as being inadequate. In this example if five or fewer households are not visited by the CHW, the performance is adequate. If six or more households are not visited, the CHW performance is considered inadequate.

Thus the LQA sample size depends on performance standards, the classification error and the number of permissible error and all of them are interrelated. A detailed theory can be found elsewhere [15].

Data was collected on paper and captured on a CHW LQAS access database by the data management team at the study central office in Lusaka. The variables analyzed were the mean performance of households % visited in each of the survey round and also the frequency of at least one health topic being discussed by the CHWs. The data was analyzed using descriptive statistics. Chi-squared tests were used for testing associations. ANOVAs were used to compare mean performance between CHWs. Each CHW performance was evaluated and assigned a performance score. Mean performance scores were computed for all the CHWs as a sum of total households visited by each CHW/total households to be sampled × 100 as determined by the data management team at the central office in Lusaka.

## Results

### CHW household visitation coverage

The household visits by the CHW were an important task that needed to be monitored by the CHW Supervisors. The LQAS shows that the household coverage was adequate in most of the rounds except in rounds 1 and 7 were the performance was inadequate (five of the 33 CHW) at two primary care facilities and needed improvement and mentorship.

The 33 zones had a total of 4,616 households. Each household was visited every quarter, resulting in 32,212 visits during the 7 surveys. Between 355 and 558 households were visited during each round of the LQAS survey, and CHW supervisors performed an average of 24 LQAS visits following each survey round. The mean performance of the CHWs was 94.9% (range 89.0–97.9%); round 1 had the lowest scores while round 6 had the highest scores as summarized in Table 2. There was no empiric evidence of the CHWs not visiting the households as the 75% benchmark was always met; rather, all sites trended over rounds toward increased CHW performance (*p* = 0.0014).

**Table 2:** Summarized LQAS results in Luangwa District

Site	Zone	Round 1			Round 2			Round 3			Round 4		
	# of Zones	Target	achieved	Classification	Target	Achieved	Classification	Target	Achieved	Classification	Target	Achieved	Classification
Mandombe	4	76	76	success	76	76	success	76	76	success	76	75	success
Luangwa Boma	6	76	73	success	76	69	success	76	71	success	76	73	success
Mphuka	5	95	95	success	76	74	success	95	90	success	76	57	failure
Kasinsa	5	76	68	success	76	67	success	95	88	success	76	53	failure
Luangwa High Sch.	4	76	43	failure	76	72	success	76	71	success	76	73	success
Sinyawagora	4										76	67	success
Chitope	6												
Totals	34		355			358			392			398	
# CHWs Sampled & validated.			21			20			22			22	
Site	Zone	Round 5			Round 6			Round 7					
	# of Zones	Target	Achieved	Classification	Target	Achieved	Classification	Target	Achieved	Classification			
Mandombe	4	76	75	success	76	76	success	76	76	success			
Luangwa Boma	6	76	73	success	76	71	success	76	76	success			
Mphuka	5	76	57	failure	95	95	success	95	79	success			
Kasinsa	5	76	53	failure	95	90	success	95	93	success			
Luangwa High Sch.	4	76	73	success	76	75	success	76	76	success			
Sinyawagora	4	76	67	success	76	76	success	76	75	success			
Chitope	6				76	75	success	76	76	success			
Totals	34	456	398		570	558		570	551				
# CHWs Sampled & validated.			22			30			30				

No difference in performance was found among the CHWs when their sex or educational attainment level was considered. Of note, at one site where one CHW attained only grade 9 level, LQAS scores were consistently as high as those in another site where all the CHWs had reached grade 12.

### Health topics discussed

**Fig. 1:**
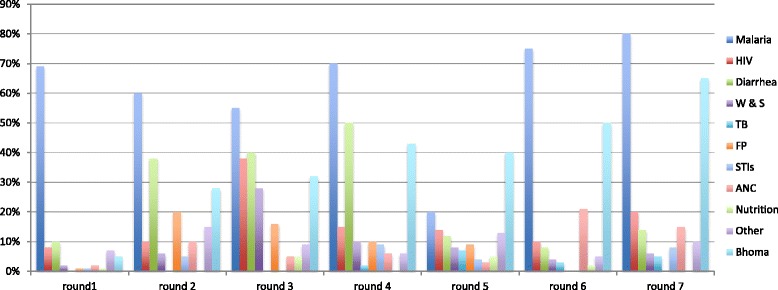
Frequency of topics discussed for each round

Households reported the following health education topics being discussed by CHWs during their visits in descending order: malaria, HIV/AIDS, other, diarrhea, family planning, water and sanitation, TB, child health, STIs and pregnancy. Malaria was the most discussed topic in every round as seen in Fig. 1 (Health topic discussed per round by CHWs). This is attributed to the high incidence of malaria cases recorded in most of the primary care facilities and also the district being in a valley and surrounded by two rivers.

## Discussion

LQAS provided an objective assessment of CHW performance regarding household visitation rate and confirmation of key health information delivered at the household level. In this study, LQAS demonstrated consistent household visits by CHWs and hence validated its applicability in our settings for monitoring CHW performance. This finding is consistent with previous studies that have used LQAS to monitor CHW performance [18]. Two major additional findings from this study were (1) the confirmed CHW household visitation rate demonstrated a positive trend, increasing over time, and (2) the pattern of topics discussed was consistent with the burden of disease as captured through the OPD attendances at the health facility and common illnesses that were identified in the CHW zones. LQAS improved accountability of CHWs (through the requirement of providing information on their performance) and enabled continuous feedback on their performance. Through both pathways, LQAS proved an important mechanism for monitoring household visitations.

In low- and middle-income settings, CHWs play a critical role in health care provision, and objective assessment of their performance is key. This is particularly important in countries like Zambia where the government is expanding involvement of CHWs through a training programme that results in a formal civil service job such as the Community Health Assistant (CHA) [6].

District planners can evaluate CHW data quality through LQAS to identify targeted priority areas for investing resources [17, 19, 20]. LQAS was easy to implement as it did not require complicated epidemiological or statistical designs and was manageable in the field. LQAS also proved to be a powerful performance appraisal tool offering project supervisors a method of identifying both well- and poorly-performing workers and tracking their performance improvements [19–21].

In the BHOMA project, the opportunity for routine feedback on performance following each round was found to be a key improvement factor; CHWs understood well the use of the monitoring tool and were aware that poor performance would not go unnoticed. This finding is consistent with previous literature demonstrating that LQAS as a monitoring tool results in improved health outputs and outcomes including underperforming areas [22–24].

A limitation of our study was that we were not able to objectively assess any correlations between improved CHW performance and improvements in the quality of care at individual PHCs or any impact on morbidity or mortality, which were the key objectives of the main project. With an objective impact assessment, the effectiveness of CHWs should also be conducted with the LQAS methodology providing useful individual and zonal level performance data. These data are likely to be essential for informing any national scale up considerations.

## Conclusion

Our results demonstrate that supervisors can feasibly monitor CHW performance through regular LQAS surveys. This methodology is not complicated to design, implement, or monitor as demonstrated by its application in one of the most rural and hard-to-reach areas of Zambia. We recommend its broader application for validating surveys and routine healthcare programmes.

## Additional file

Additional file 1: BHOMA household monitoring tool. (PDF 90 kb)

## Abbreviations

AIDS: Acquired Immuno Deficiency Syndrome
ANOVA: Analysis of Variation
BHOMA: Better Health Outcomes through Mentoring and Assessments
CHA: Community health assistant
CHW: Community health worker
CIDRZ: Centre for Infectious Disease Research in Zambia
HIV: Human immunodeficiency virus
ID: Identity number
IMCI: Integrated management of childhood illnesses
LQAS: Lot quality assurance sampling
NGO: Non-governmental organization
NHC: Neighbourhood health committee
OPD: Outpatient department
PHC: Primary health care
STI: Sexually transmitted infections
TB: Tuberculosis
TBA: Traditional birth attendants
