# Supplementary material for: Use of Lot quality assurance sampling surveys to evaluate community health worker performance in rural Zambia: a case of Luangwa district
Source: BMC Health Serv Res. 2017 Apr 17;17:279. doi: 10.1186/s12913-017-2229-9 (PMC5393033; doi:10.1186/s12913-017-2229-9)
Supplement: Additional file 1: — BHOMA household monitoring tool. (PDF 90 kb) [file 12913_2017_2229_MOESM1_ESM.pdf]

BHOMA HOUSEHOLD MONITORING TOOL

**District:**	**CHW's Name:**	**Number of HH in Zone:**
**Health Facility:**	**CHW's Zone:**	**Date of Survey:**

**1. No Of HH Sampled:**	**2. Are you the head of this Household?** ☐ Yes ☐ No	**3. Sex of Respondent:** ☐ Male ☐ Female	**4. Does anyone in this HH know about BHOMA?** ☐ Yes ☐ No	**5. Has this HH Been visited by a BHOMA CHW in the last 4 months?** ☐ Yes ☐ No ☐Not Sure	**6. Has BHOMA helped change your household's health seaking behavior?** ☐ Yes ☐ No	**7. Average time a CHW spends at your house:** ☐ Short ☐ Medium ☐ Long ☐ N/A	**8. Topics discussed with BHOMA CHW at last visit (tick all that apply)**
**9. Comments:**							☐Malaria ☐HIV/AIDS ☐STI ☐Pregnancy ☐Diarrhea ☐Child Health ☐BHOMA ☐Nutrition ☐Water/Sanitation ☐TB ☐Family Planning ☐Other ☐N/A

**1. No Of HH Sampled:**	**2. Are you the head of this Household?** ☐ Yes ☐ No	**3. Sex of Respondent:** ☐ Male ☐ Female	**4. Does anyone in this HH know about BHOMA?** ☐ Yes ☐ No	**5. Has this HH Been visited by a BHOMA CHW in the last 4 months?** ☐ Yes ☐ No ☐Not Sure	**6. Has BHOMA helped change your household's health seaking behavior?** ☐ Yes ☐ No	**7. Average time a CHW spends at your house:** ☐ Short ☐ Medium ☐ Long ☐ N/A	**8. Topics discussed with BHOMA CHW at last visit (tick all that apply)**
**9. Comments:**							☐Malaria ☐HIV/AIDS ☐STI ☐Pregnancy ☐Diarrhea ☐Child Health ☐BHOMA ☐Nutrition ☐Water/Sanitation ☐TB ☐Family Planning ☐Other ☐N/A

**1. No Of HH Sampled:**	**2. Are you the head of this Household?** ☐ Yes ☐ No	**3. Sex of Respondent:** ☐ Male ☐ Female	**4. Does anyone in this HH know about BHOMA?** ☐ Yes ☐ No	**5. Has this HH Been visited by a BHOMA CHW in the last 4 months?** ☐ Yes ☐ No ☐Not Sure	**6. Has BHOMA helped change your household's health seaking behavior?** ☐ Yes ☐ No	**7. Average time a CHW spends at your house:** ☐ Short ☐ Medium ☐ Long ☐ N/A	**8. Topics discussed with BHOMA CHW at last visit (tick all that apply)**
**9. Comments:**							☐Malaria ☐HIV/AIDS ☐STI ☐Pregnancy ☐Diarrhea ☐Child Health ☐BHOMA ☐Nutrition ☐Water/Sanitation ☐TB ☐Family Planning ☐Other ☐N/A

Sampled by:

Signature:

Household sampling tool v1.1
